# Supplementary material for: A thermoplastic chip for 2D and 3D correlative assays combining screening and high-resolution imaging of immune cell responses
Source: Cell Rep Methods. 2025 Jan 17;5(1):100965. doi: 10.1016/j.crmeth.2025.100965 (PMC11841093; doi:10.1016/j.crmeth.2025.100965)
Supplement: Document S1. Figures S1–S6 and Table S1 [file mmc1.pdf]

Cell Reports Methods, Volume *5*

Supplemental information

A thermoplastic chip for 2D and 3D correlative assays combining screening and high-resolution imaging of immune cell responses

Hanna van Ooijen, Quentin Verron, Hanqing Zhang, Patrick A. Sandoz, Thomas W. Frisk, Valentina Carannante, Karl Olofsson, Arnika K. Wagner, Niklas Sandström, and Björn Önfelt

Supplemental information to:

A thermoplastic chip for 2D and 3D correlative assays combining screening and high-resolution imaging of immune cell responses

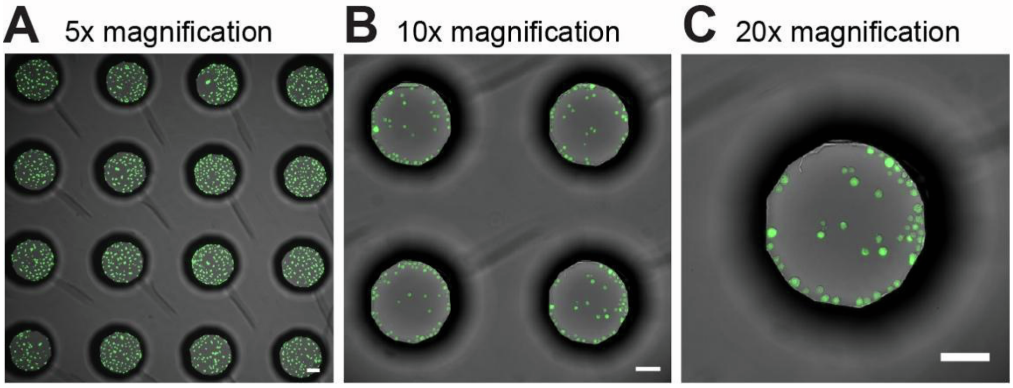

Supplemental figure S1. A microwell array optimized for wide-field microscopy. Related to Figure 1. (**A-C)** Example wide-field microscopy images of the region of the chip fitting within the FoV using a 5x objective (A), a 10x objective (B) or a 20x objective (C), and a camera sensor of 13x13 mm^2. Scale bars: 100 µm.

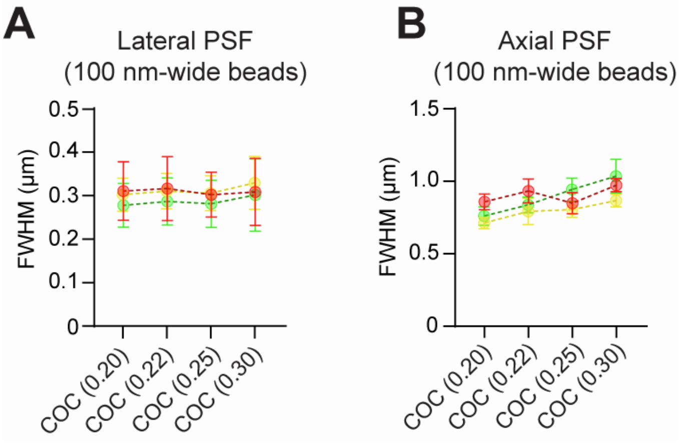

Supplemental figure S2. Effect of the bottom thickness on the optical performance of the chip. Related to Figure 2. **(A-B)** Sub-diffraction 100 nm-wide beads seeded in the chip microwells were imaged with an oil-immersion 63x/1.4 objective. Width of the lateral (A) and axial (B) PSF. Pooled data from 3 independent experiments, with a total of n=94-129 beads per condition.

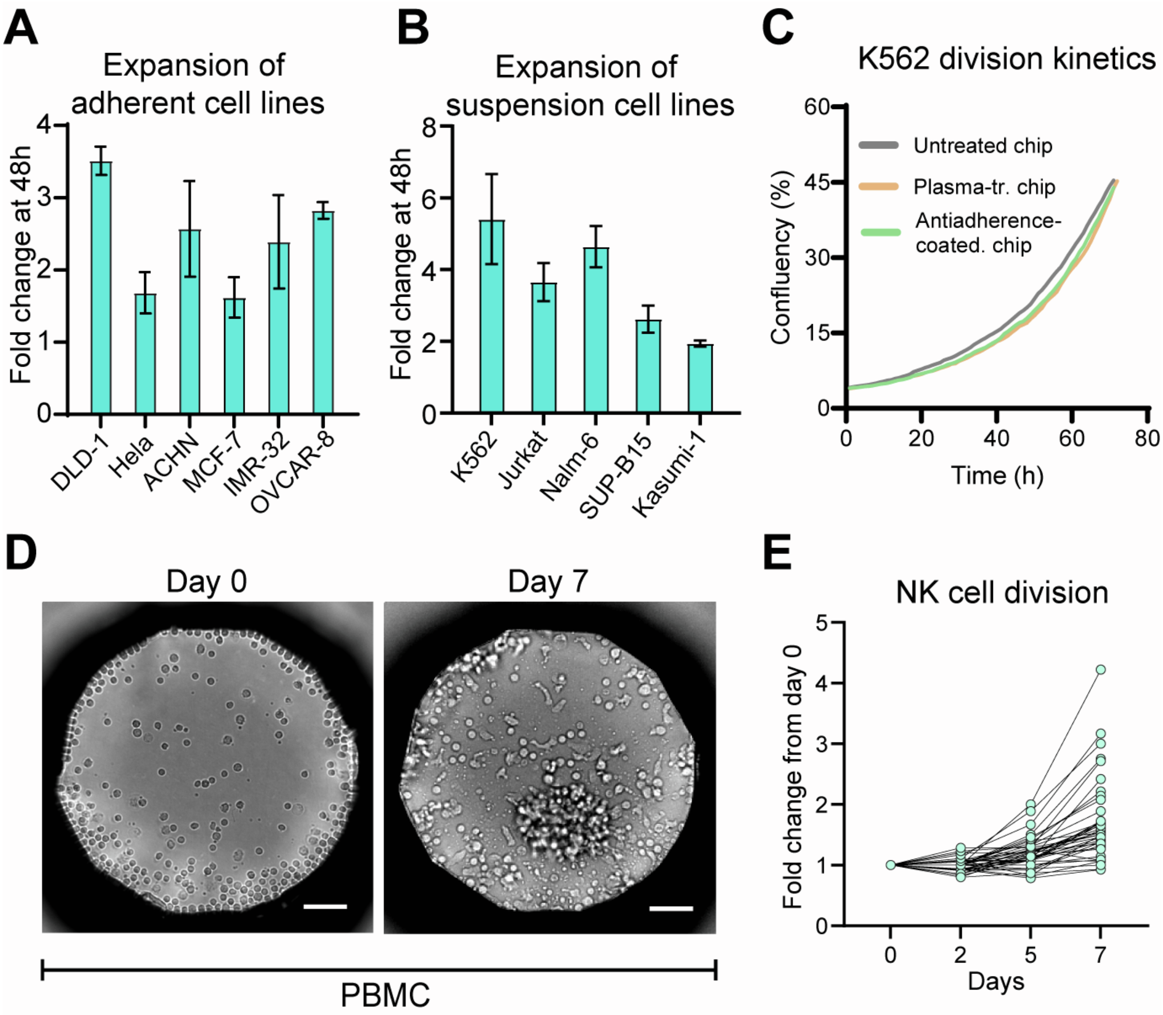

Supplemental figure S3. 2D cell division in the microwell chip. Related to Figure 3. **(A)** Division rate of a range of adherent cell lines grown in oxygen plasma-treated microwells. **(B)** Division rate of a range of suspension cell lines grown in anti-adherence-treated microwells. Each bar in (A-B) represents the average of 2-5 wells with corresponding standard deviation. **(C)** Confluency over time, for K562 cells grown in microwells with different surface treatments. **(D)** Example images of PBMCs grown for 7 days in anti-adherence-treated microwells. Scale bars: 50 µm. **(E)** Division kinetics of primary NK cells grown for 7 days in anti-adherence-treated microwells.

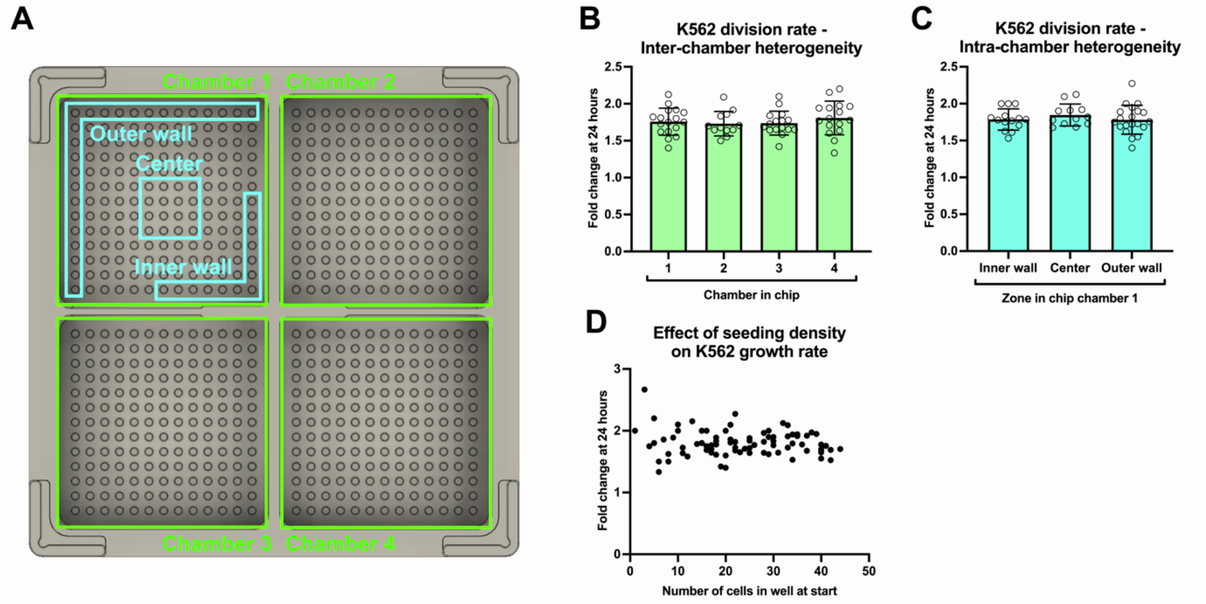

Supplemental figure S4. Cell proliferation across the microwell chip. Related to Figure 3. K562 tumor cells were cultured in the microwells, and their growth rate was related to the well location on the chip. **(A)** The microwell array, composed of 4 chambers, was further divided into regions of interest with possibly different environmental conditions. **(B-C)** Growth rate of K562 cells over 24 hours, compared between chambers 1-4 (B) or between regions of a single chamber (C). **(D)** Correlation between the average K562 growth rate in microwells and the number of cells at the start of the experiment. Each bar in (B-C) indicates the average of at least 8 microwells, while each dot in (B-D) represents a single microwell. Only wells containing at least 5 cells were included in the analysis in (B-C).

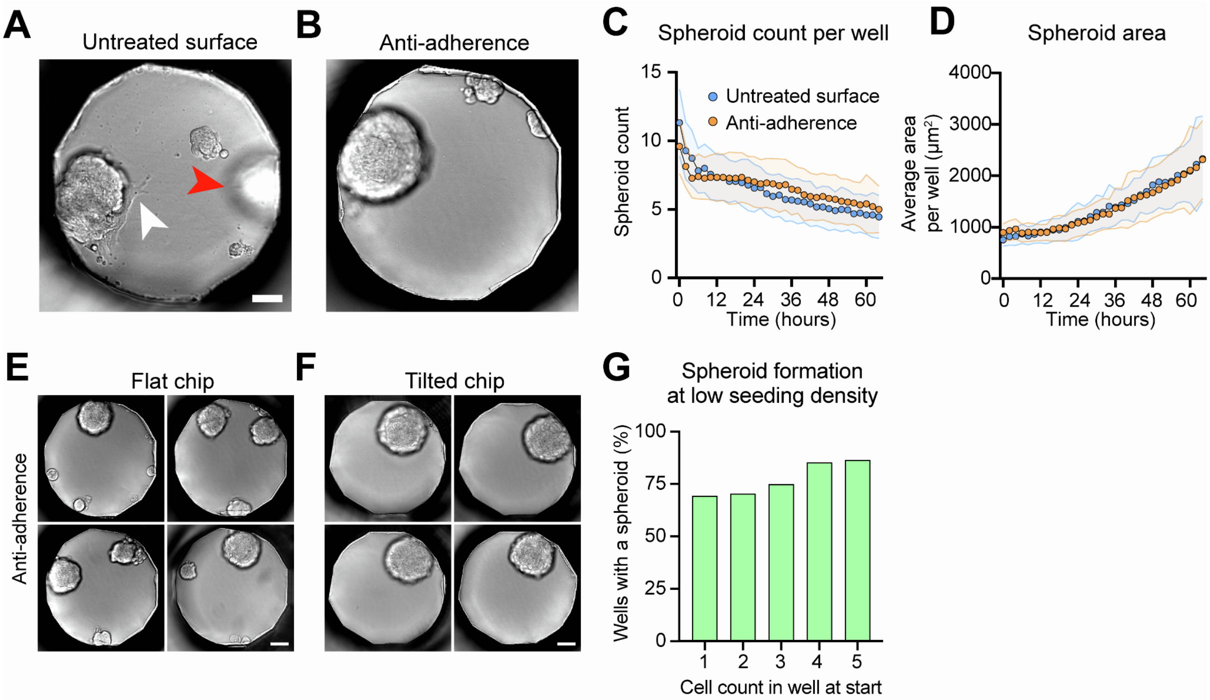

Supplemental figure S5. Comparison of protocols to form spheroids in the microwells. Related to Figure 5. (A-B) Example bright-field images of DLD-1 spheroids formed in untreated (A) and anti-adherence-treated (B) microwells. White arrowhead: protrusions binding a spheroid to the untreated surface of the well. Red arrowhead: spheroid displaced to an out-of-focus plane. (C-D) Comparison of the growth kinetics of spheroids in untreated and anti-adherence-treated chips, considering the average number of cell clusters per well (C) and their mean area (D). (E-F) Representative bright-field images of the spheroids obtained by either keeping the chip in a horizontal position (E) or by tilting it slightly (F) during spheroid growth. Scale bars: 50 μm (G) Fraction of wells containing a spheroid at day 7, for a given number of DLD-1 cells in the well at seeding.

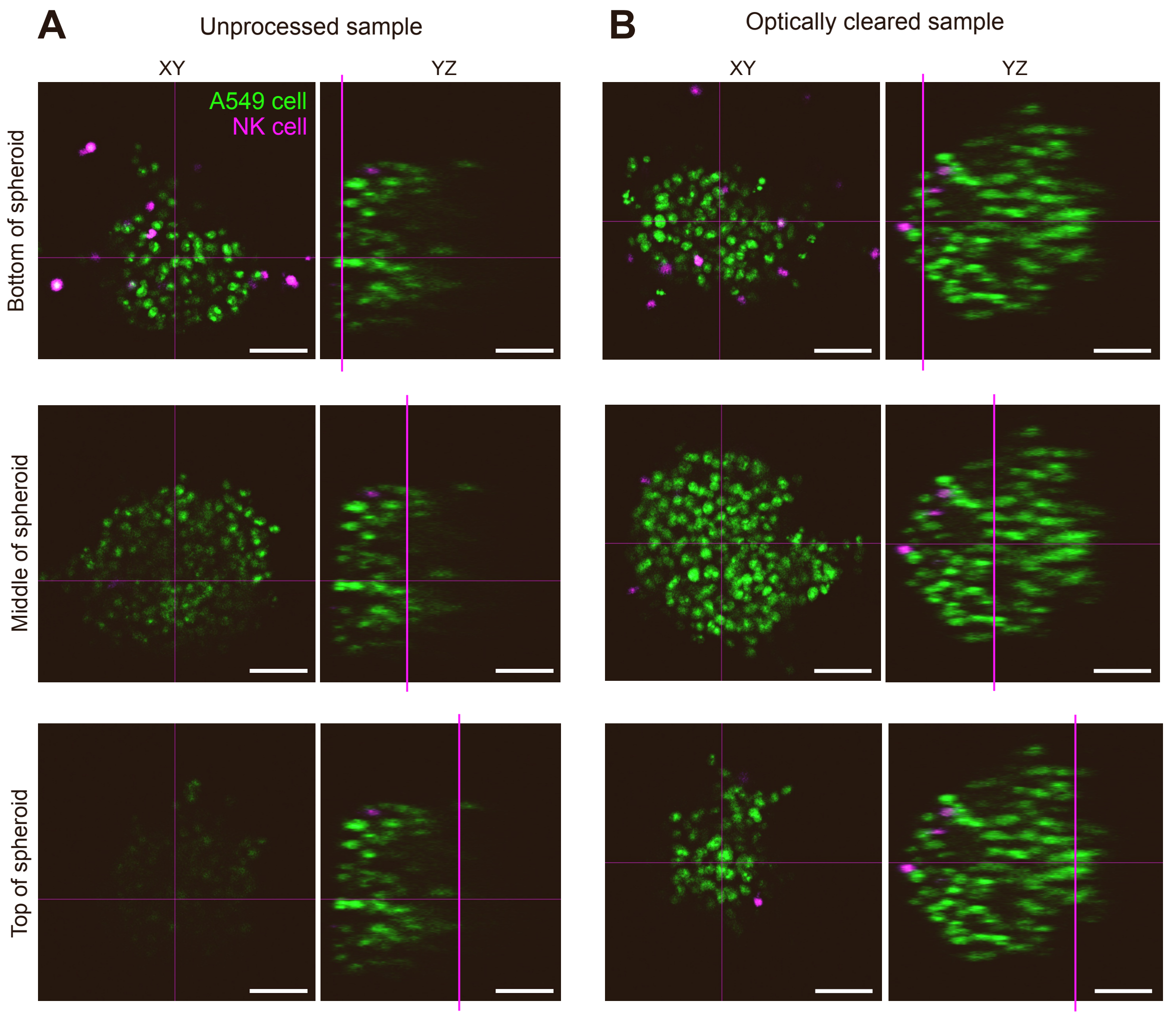

Supplemental figure S6. Improvement to deep imaging of spheroids by optical clearing. Related to Figure 5. (A-B) Co-cultures of A549 spheroids and NK cells were fixed after 16 hours and either directly imaged with confocal microscopy (A), or first, permeabilized and embedded in a refractive index-matched medium before imaging (optical clearing) (B). The magenta cross indicates the cutting planes for the XY and YZ images. All scale bars 50 µm.

Supplemental Table 1. Spheroid formation in microwells using a range of immortal cell lines. Related to Fig. 5.

Cell line	Tissue	Cell type	Makes spheroids
DLD-1	Colon	Epithelial	yes
HCT116	Colon	Epithelial	yes
SW620	Colon	Epithelial	yes
IMR-90	Lung	Fibroblast	yes
MRC-5	Lung	Fibroblast	yes
WI-38	Lung	Fibroblast	yes
A549	Lung	Epithelial	yes
A-498	Kidney	Epithelial	yes
ACHN	Kidney	Epithelial	no
HeLa	Cervix	Epithelial	yes
IMR-32	Brain	Neuroblast	yes
OVCAR-8	Ovary	Epithelial	yes
MCF-7	Breast	Epithelial	yes
BJ	Skin	Fibroblast	yes
